# *Deladenus bonabensis* n. sp. (Tylenchomorpha: Neotylenchidae) from East Azarbaijan province, northwestern Iran: A morphological and molecular phylogenetic study

**DOI:** 10.2478/jofnem-2023-0002

**Published:** 2023-03-09

**Authors:** Mohammad Amiri Bonab, Mohammad Reza Atighi, Majid Pedram

**Affiliations:** Department of Plant Pathology, Faculty of Agriculture, Tarbiat Modares University, Tehran, Iran

**Keywords:** City of Bonab, *Deladenus brevis*, New species, Taxonomy

## Abstract

A population of *Deladenus*, representing a new species, was recovered from Bonab’s Ghara-Gheshlagh lagoon. It is mainly characterized by having a long body (1051–1185 μm), long distance of anterior end to pharyngeal glands end (270–312 μm), six lines in lateral fields, and a short mucro-like differentiation on the tail tip. Furthermore, it has a small-sized stylet (8.5–11.0 μm) with three knobs, no postvulval uterine sac, and males with 24- to 28-μm-long tylenchoid spicules and penial tube. With six lateral lines, the new species is comparable with seven species of the genus: *D. apopkaetus*, *D. brevis*, *D. cocophilus*, *D. durus*, *D. obtusicaudatus*, *D. processus*, and *D. ulani*. It was furthermore compared with *D. oryzae* with an unknown number of lateral lines and *D. aridus*, *D. obesus*, and *D. parvus* having a different number of lateral lines but similar morphology. In the molecular phylogenic analyses using small and large subunit ribosomal DNA (SSU and LSU D2-D3 rDNA) sequences, the relationships of the new species with other species and genera were not resolved in SSU phylogeny. However, it formed a clade with *Deladenus* sp. and *D. brevis* in LSU phylogeny.

The genus *Deladenus* was established by Thorne (1941), describing *D. durus* (Cobb, 1922) Thorne, 1941 as its type species. It was placed in the subfamily Neotylenchinae Thorne, 1941 (it is now under the family Neotylenchidae Thorne, 1941 *sensu* Siddiqi, 2000). Compared to other genera in the family Neotylenchidae, it is characterized by low lip reign, small stylet, anteriorly located pharyngo-intestinal junction to the nerve ring, secretory-excretory pore (S-E pore) at the level or slightly behind the nerve ring, no median bulb in pharynx, long dorsal overlap of pharyngeal glands over intestine, no postvulval uterine sac (PUS), and males with bursa. The genus has two free-living mycetophagous and entomoparasitic generations (Siddiqi, 2000). The free-living phase has a typical neotylenchoid life cycle, and the entomoparasitic phase has a typical sphaerularid cycle (Miraeiz et al., 2017). Currently, around 14 species of the genus are known by their mycetophagous phase. The list of valid species of the genus has already been given by several authors (Siddiqi, 2000; Miraeiz et al., 2017; Yu et al., 2017; Morris et al., 2018). The two species *D. gilanica* Esmaeili, Jalalinasab, Ye, & Heydari, 2020 and *D. brevis* Heydari, Abolafia, & Pedram, 2020 are the most recent species added to the genus (Esmaeili et al., 2020; Heydari et al., 2020).

In Iran, the genus was first recorded by reporting *D. durus* (Jahanshahi Afshar et al., 2014). Afterward, the diversity of the genus was enriched by describing *D. persicus* Miraeiz, Heydari & Golhasan, 2017, *D. gilanica*, and *D. brevis*, all recovered from northern Iran (Miraeiz et al., 2017; Esmaeili et al., 2020; Heydari et al., 2020). Herein, *Deladenus bonabensis* n. sp. recovered from East Azarbaijan province in northwestern Iran is described based on morphological and molecular data.

## Materials and Methods

### Soil sampling, nematode extraction, and morphological identification

Several soil samples were collected from different parts of the city of Bonab, East Azarbaijan province, northwestern Iran. The tray method (Whitehead and Hemming, 1965) was employed to extract nematodes from soil samples. The nematodes of interest were hand-picked under a Nikon SMZ1000 stereomicroscope, heat-killed by adding boiling 4% formaldehyde solution, transferred to anhydrous glycerin (De Grisse, 1969), mounted on permanent slides, and examined using a Nikon Eclipse E600 light microscope. Photographs were taken using an Olympus DP72 digital camera attached to an Olympus BX51 microscope powered with differential interference contrast. Drawings were made using a drawing tube attached to the microscope and were redrawn using CorelDRAW^®^ software version 16.

### DNA extraction, PCR, and sequencing

A single nematode specimen of the new species was picked out and transferred to a small drop of TE buffer (10 mM Tris-Cl, 0.5 mM EDTA; pH 9.0, Qiagen) on a clean slide and squashed using a clean coverslip. The suspension was collected by adding 35 μl of TE buffer. DNA sample was stored at −20°C until used as PCR template. Primers for partial amplification of small subunit (SSU) rDNA were forward primer F22 (5´–TCCAAGGAAGGCAGCAGGC–3´) (Dorris et al., 2002) and reverse primer 1573R (5´– TACAAAGGGCAGGGACGTAAT–3´) (Mullin et al., 2005). The large subunit (LSU) rDNA D2-D3 expansion segments were amplified using the forward D2A (5´– ACAAGTACCGTGAGGGAAAGTTG–3´) and reverse D3B (5´–TCGGAAGGAACCAGCTACTA–3´) primer pairs (Nunn, 1992). The PCR products were sequenced in both directions using the same primers used in PCR with an ABI3730XL sequencer. The newly obtained sequences were deposited into the GenBank database under the accession numbers OP250137 for SSU and OP250136 for LSU rDNA sequences.

The newly obtained sequences were compared with those of other nematode species available in GenBank using the BLAST homology search program. For reconstruction of the phylogenetic relationships, two independent SSU and LSU data sets were prepared. The selected SSU sequences were aligned using the Q-INS-i algorithm of the online version of MAFFT (version 0.91b) (see http://mafft.cbrc.jp/aligment/seaver/; Katoh and Standley, 2013). Clustal X2 was used to align the LSU sequences (Larkin et al., 2007). The poorly aligned positions and divergent regions of SSU and LSU data sets were eliminated using Gblocks (see http://phylogeny.lirmm.fr/phylo_cgi/one_task.cgi?task_type=gblocks) and selecting all three less stringent options. The model of base substitution was selected using MrModeltest.2 (Nylander, 2004). The Bayesian analyses were performed using MrBayes v3.1.2 (Ronquist and Huelsenbeck, 2003) running the chains for 5 million generations for both data sets. After discarding burn-in samples, the remaining samples were retained for further analyses. The Markov chain Monte Carlo method within a Bayesian framework was used to estimate the posterior probabilities of the phylogenetic trees using the 50% consensus majority rule (Larget and Simon, 1999). Adequacy of the posterior sample size was evaluated using autocorrelation statistics as implemented in Tracer v1.6 (Rambaut and Drummond, 2009). The species *Acrobeloides maximus* Thorne, 1925, *Acrobeles ciliatus* von Linstow, 1877, and *Pseudacrobels* sp. were used as outgroup species in SSU; and *Poikilolaimus oxycerca* de Man, 1895, *P. piniperdae* Fuchs, 1930, and *Oscheius myriophilus* Poinar, 1986 were used as outgroups in LSU phylogenies. The output files of the phylogenetic program were visualized using Dendroscope V3.2.8 (Huson and Scornavacca, 2012) and were digitally drawn in CorelDRAW® software version 16.

## Results

**Figure 1:**
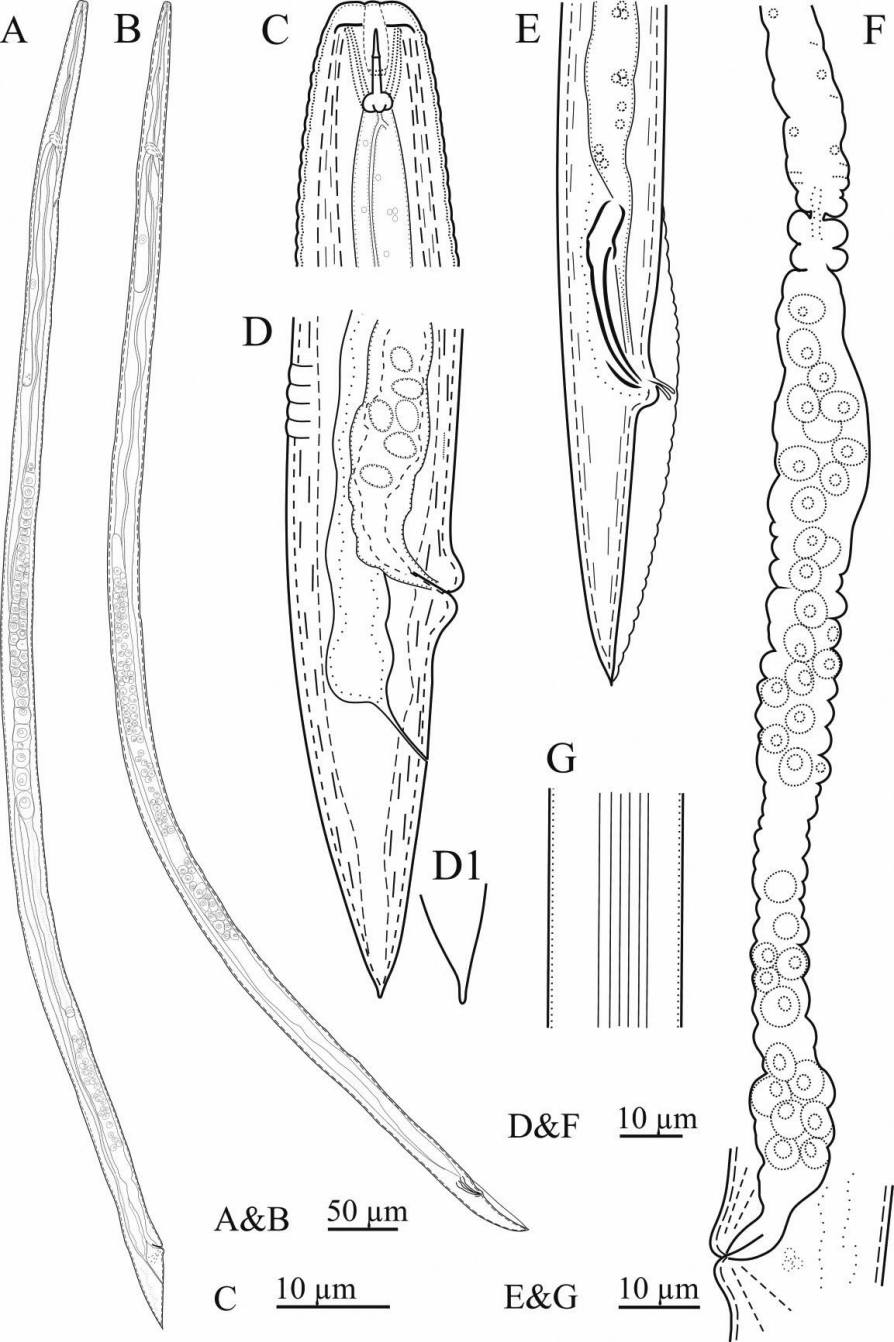
Line drawings *of Deladenus bonabensis* n. sp. A: Mycetophagous female; B: Mycetophagous male; C: Anterior body region, female; D: Posterior body region, female; D1: Tail tip, female; E: Posterior body region, male; F: Parts of reproductive system, female; G: Lateral lines, female.

**Figure 2:**
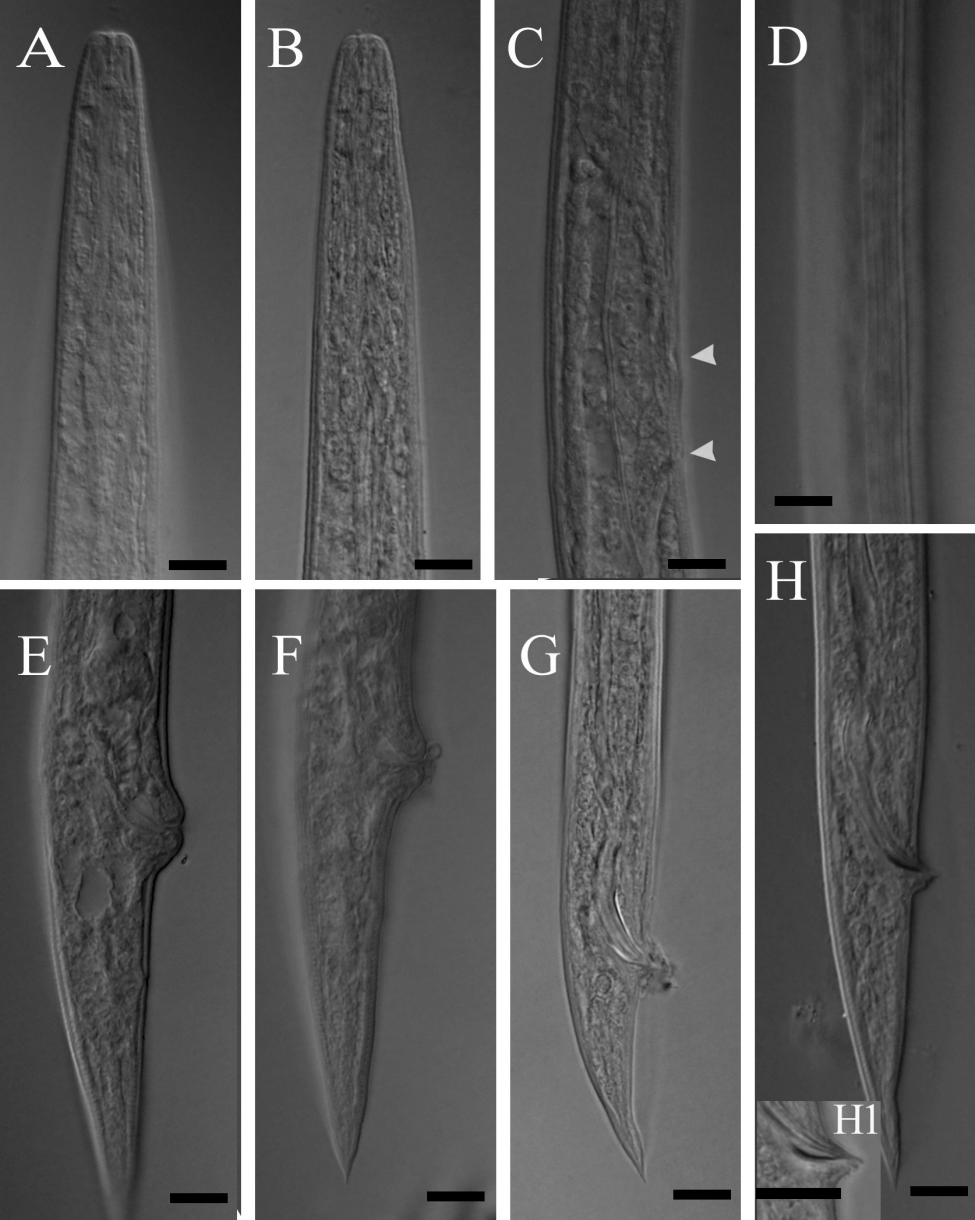
Light micrographs of *Deladenus bonabensis* n. sp. A: Anterior body region, female; B: Anterior body region, male; C: Part of pharynx, female, showing the position of secretory-excretory pore and hemizonid (upper arrow showng hemizonid, lower arrow showing the secretory-excretory pore); D: Lateral lines, female; E & F: Posterior body region, female; G: Posterior body region, male; H: Spicules and gubernaculum, male; H1: Penial tube, male. All scale bars = 10 μm.

*Deladenus bonabensis* n. sp. (Table 1; Figs. 1 and 2).

**Table 1:** Morphometrics of *Deladenus bonabensis* n. sp. All measurements are in μm and in the form: mean ± standard deviation (range).

Characters	Holotype	Paratypes	

	Female	Females	Males
n	1	9	8
L	1085	1088 ± 45 (1051–1185)	986 ± 32 (940–1039)
a	42.7	42.7 ± 3.0 (39–48)	46.5 ± 5.5 (38.5–57.5)
b	9.0	9.0 ± 0.5 (8.0–9.5)	8.8 ± 0.5 (8.5–9.5)
b´	3.7	3.8 ± 0.2 (3.5–4.0)	4.0 ± 0.3 (3.8–4.7)
c	27.0	27.0 ± 2.5 (23.5–29.5)	26.0 ± 4.0 (20.5–33.5)
c´	2.3	2.5 ± 0.3 (2.0–3.0)	2.5 ± 0.3 (2.2–3.0)
V or T	94.5	94.2 ± 0.5 (93.5–95.0)	52.5 ± 3.5 (48.5–58.5)
Lip region height	2	2.3 ± 0.2 (2.0–2.5)	2.5 ± 0.1 (2.2–2.5)
Lip region width at base	7.5	7.3 ± 0.7 (6–8)	7.0 ± 0.3 (6.5–7.5)
Lip region width at the apex	5.7	5.5 ± 0.5 (5.0–6.5)	5.5 ± 0.5 (5–6)
Max. body dim.	26	25.5 ± 2.0 (22–28)	21.5 ± 3.0 (17–27)
Pharynx	124	123 ± 7 (114–135)	112 ± 6 (101–122)
Anterior end to the end of pharyngeal glands	272	289 ± 18 (270–312)	243 ± 21 (205–267)
Reproductive tract	683	655 ± 41 (574–705)	517 ± 33 (488–561)
Anterior end to vulva	1021	1025 ± 44 (982–1120)	—
Stylet total length	9	9.5 ± 1.0 (8.5–11.0)	9.5 ± 0.5 (9.0–10.5)
Stylet conus	4	4.0 ± 0.5 (3.5–5.0)	4.0 ± 0.5 (3.5–4.5)
Anterior end to DGO	11.5	12.0 ± 1.0 (11.0–13.5)	—
Secretory-excretory pore from anterior end	158	163 ± 11 (147–181)	149 ± 17 (134–185)
Nerve ring from anterior end	112	116 ± 7 (108–127)	112 ± 14 (97–140)
Hemizonid from anterior end	148	148 ± 6 (140–159)	137 ± 19 (119–178)
Vulval body diameter	27	25 ± 2 (21–28)	—
Anal body diameter	18	17.0 ± 1.5 (15–19)	15.0 ± 1.5 (13–18)
Tail	40	40.5 ± 3.5 (36–46)	39.0 ± 6.5 (30–51)
Spicules	—	—	25.5 ± 1.5 (24–28)
Gubernaculum	—	—	7.0 ± 0.5 (7–8)

### Description

*Free-living mycetophagous female*: These are medium-size nematodes, with a slender body, gradually narrowing toward the anterior body end. Six lines are present in lateral fields. The lip reign is low, not set off from the rest of the body.

The stylet is small, with three knobs at base, its shaft longer than conus. The dorsal gland orifice opens at the base of the stylet knobs. Hemizonid is at the level of the nerve ring. The S-E pore is posterior to hemizonid. A deirid is not seen. Pharynx with no median bulb, and its junction with intestine is anterior to the nerve ring. The dorsal pharyngeal gland is long, overlapping intestine. The intestine is simple, and the rectum and anus are functional. The reproductive system is monodelphic-prodelphic, with ovary outstretched, oocytes in single or double rows, oviduct tubular, spermatheca oblong-oval, filled with large sperm cells, crustoformeria with more than four cells in each row, vagina modernity sclerotized, vulva with elevated lips, no PUS, and no vulval flap. The tail is conical, uniformly narrowing to the end, with a small mucro-like differentiation at the tip.

*Infective female*: An infective female specimen was not found.

*Parasitic female*: A parasitic female specimen was not found.

*Free-living mycetophagus male*: These were present as frequently as females. General morphology and pharynx are similar to those of females. The reproductive system is monorchic. Spicules are tylenchiod. Gubernaculum is small and thin. A penial tube is present. Bursa well developed, enveloping the entire tail.

### Type habitat and locality

Specimens were recovered from a soil sample collected in Ghara-Gheshlagh lagoon, Bonab, East Azarbaijan province, northwestern Iran, in December 2019. The Global Positioning System coordinate for the locality is 37°12´29˝N 45°57´57˝E.

### Etymology

The specific name refers to the city of Bonab, where the new species was recovered.

### Type material

Holotype female, four paratype females, and four paratype males were deposited at the WaNeCo nematode collection of the Wageningen University, The Netherlands. Four paratype females and four paratype males were deposited at the nematode collection of Faculty of Agriculture, Tarbiat Modares University, Tehran, Iran.

The LSID for this publication is urn:lsid:zoobank. or g:p u b:6D91D72 A-AD3 5 - 4 5 3B-A4 3 F-459770CBB5E7.

## Diagnosis and relationships

*Deladenus bonabensis* n. sp. is mainly delimited by 1051- to 1185-μm-long females, with six lines in the lateral fields, vulva with no lateral flaps, conical tail, and penial tube present. It is further characterized by a stylet 8.5- to 11.0-μm-long with three basal knobs. By having six lateral lines, the new species is comparable to seven species of the genus, namely *D. apopkaetus* Chitambar, 1991; *D. brevis*; *D. cocophilus* Nasira, Shahina & Firoza, 2013; *D. durus*; *D. obtusicaudatus* Bajaj, 2015; *D. processus* Tomar, Somvanshi & Bajaj, 2015; and *D. ulani* Sultanalieva, 1983. It was further compared with *D. oryzae* Bajaj, 2015, with an unknown number of lateral lines, and *D. aridus* Andrássy, 1957; *D. obesus* Thorne, 1941; and *D. parvus* Zell, 1985, having a different number of lateral lines, but similar morphology. The comparisons with the aforementioned species follow.

*Deladenus bonabensis* n. sp. is distinguished from *D. apopkaetus* by its longer body (1051–1185 vs. 612–840 μm), absence of pharyngeal chamber (vs. its presence), shorter stylet (8.5–11.0 vs. 11.9– 13.5 μm) and S-E pore posterior to hemizonid (vs. well anterior to hemizonid) with a simple (vs. cuticularized) duct.

The new species is distinguished from *D. brevis* by longer females and males (total range) (940–1185 vs. 367–454 μm), no flap on the vulva (vs. its presence), a mucro-like differentiation at the tail tip (vs. not), a longer stylet (8.5–11.0 vs. 6–7 μm), greater V value (93.5–95.0 vs. 87.2–90.3), and longer spicules (24–28 vs. 11.3–14.5 μm) and gubernaculum (7–8 vs. 3.4–4.5 μm).

*D. bonabensis* n. sp. differs from *D. cocophilus* in its longer females (1051–1185 vs. 550–930 μm), S-E pore after hemizonid (vs. hemizonid after S-E pore) at 147- to 181-μm distance from anterior end (vs. 65.6–84.0 μm), longer tail (36–46 vs. 22.4–28.0 μm), longer spicules (24–28 vs. 16–18 μm) and gubernaculums (7–8 vs. 4.0–5.6 μm), and tail tip shape (having a mucro-like differentiation vs. widely rounded).

It is distinct from *D. durus* (data according to Chitambar, 1991) by six vs. six or seven lines in lateral field, absence of pharyngeal chamber (vs. presence), weakly deveopled metacorpus (vs. developed) and S-E pore far from hemizonid (vs. close).

*Deladenus bonabensis* n. sp. is distinguished from *D. obtusicaudatus* mainly by its tail shape (conical, having a mucro-like differentiation at the tip vs. short and cylindrical with a broadly rounded to truncated terminus), S-E pore posterior to hemizonid (vs. anterior), and longer females (1051–1185 vs. 730 μm).

Compared with *D. processus*, *D. bonabensis* n. sp. is characterized by longer females (1051–1185 vs. 760–990 μm), higher c value (23.5–29.5 vs. 19.6–22.8), greater V value (93.5–95.0 vs. 92.2– 93.5), longer stylet (8.5–11.0 vs. 6–7 μm), and longer spicules (24–28 vs. 15–16 μm) and gubernaculums (7–8 vs. 3–4 μm).

*Deladenus bonabensis* n. sp. is distinguished from *D. ulani* by longer females (1051–1185 vs. 676– 787 μm), greater c value (23.5–29.5 vs. 20.0–21.0), absence of PUS (vs. presence), greater V value (93.5–95.0 vs. 82.0–83.0), and tail tip shape (having a mucro-like differentiation vs. bluntly rounded).

The new species differs from *D. oryzae* with a not obese (vs. obese) body (a = 39–48 vs. 25–26), a longer (1051–1185 vs. 660–960 μm) body, S-E pore posterior to hemizonid (vs. anterior), longer spicules (24–28 vs. 16–18 μm), and tail tip shape (having a mucro-like differentiation vs. bluntly rounded).

*Deladenus bonabensis* n. sp. is dinstinguished from *D. aridus* (data from Andrássy, 2007), by its longer body (1051–1185 vs. 700–880 μm), six (vs. four) lines in the lateral field, greater a value (39–48 vs. 31–37), greater c value (23.5–29.5 vs. 18.0–20.0), and greater V value (93.5–95.0 vs. 91.0–92.0).

Compared with *D. obesus* (data from the original description), *D. bonabensis* n. sp. is characterized by a longer stylet (mean = 9.5 vs. 7 μm), greater a value (39–48 vs. 10–16), greater b value (8.0–9.5 vs. 5.0–6.0), lower V value (93.5–95.0 vs. 95–99), longer distance between vulva and anus (vs. very short) and six (vs. eight to ten) lines in the lateral field according to Chitambar (1991).

*Deladenus bonabensis* n. sp. is dinstinguished from *D. parvus* by its longer body (1051–1185 vs. 388–600 μm), six (vs. four) lines in the lateral field, and greater V value (93.5–95.0 vs. 79.8–85.4).

## Molecular profiles and phylogenetic status

Sequencing of SSU and LSU rDNA D2-D3 fragments of the new species yielded a single partial SSU 908 nucleotides long, and an LSU D2-D3 sequence 674 nucleotides long. The BLAST search using the SSU sequence revealed that it has 98.79% (three mismatches) and 98.68% (12 mismatches and nine gaps) identity with two sequences assigned to Tylenchidae sp. (LC382046 and LC382045, respectively). Its identity with all other SSU sequences was less than 98%. The BLAST search using the LSU sequence revealed it has a 99% (six mismatches and two gaps) identity with a sequence assigned to Sphaerularioidea sp. (MW577121). The identity with currently available LSU sequences of relevant genera was less than 90%.

**Figure 3:**
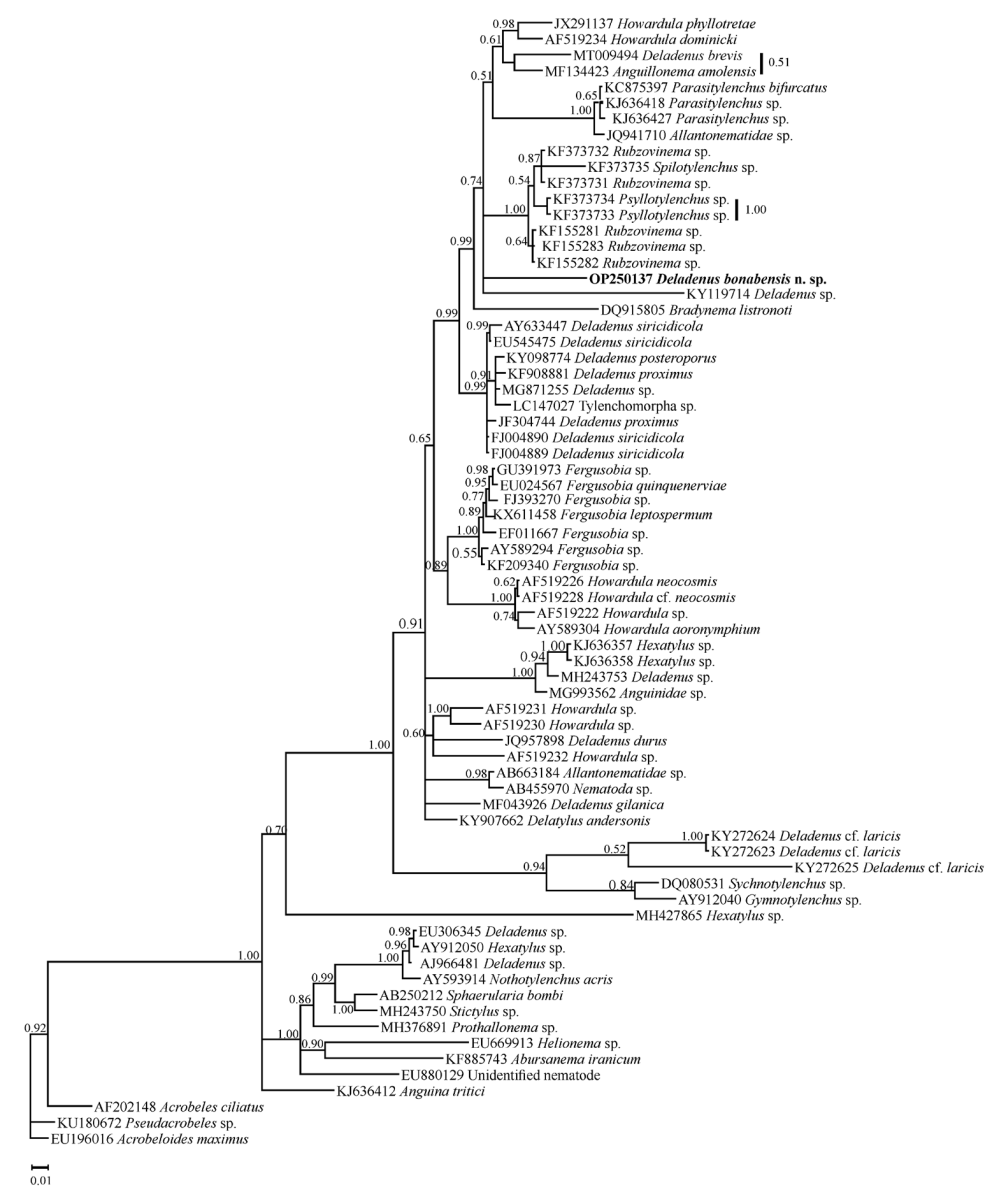
Bayesian 50% majority rule consensus tree of *Deladenus bonabensis* n. sp. based on small subunit (SSU) rDNA sequences under GTR + I + G model. Bayesian posterior probability values more than 0.50 are given for appropriate clades. The new sequence is indicated in bold.

Seventy-one sequences (including the newly generated sequence of the new species and three sequences of classic rhabditids as outgroups) were used in SSU phylogeny (species names and accession numbers are available in the SSU tree). Their alignment included 1,761 characters, 658 of which were variable. Fig. 3 represents the phylogenetic tree reconstructed using this data set. The sequences of *Deladenus* species occupied different placements in this tree and the relationships of the newly generated SSU sequence of the new species with other sequences were not resolved due to polytomy.

**Figure 4:**
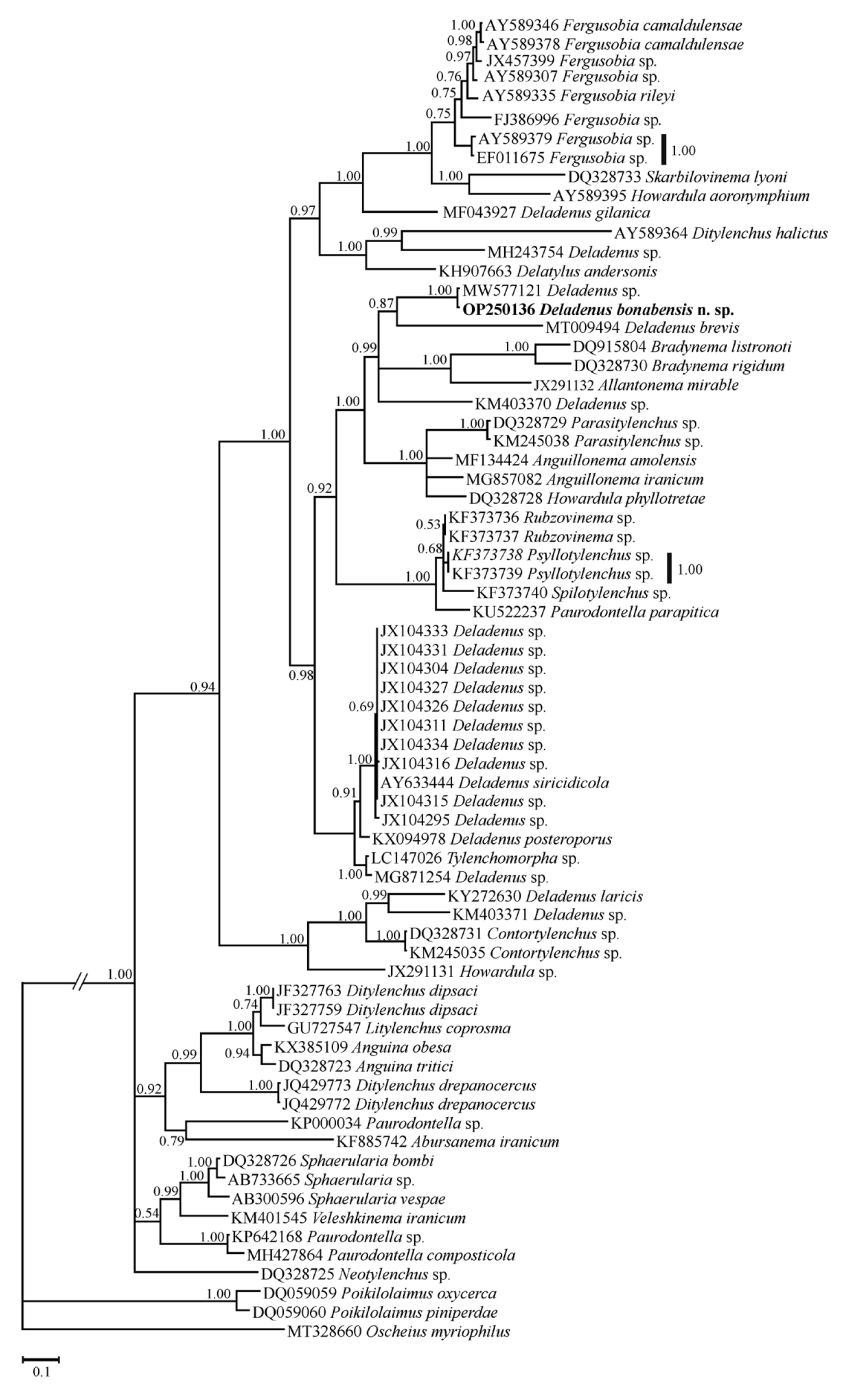
Bayesian 50% majority rule consensus tree of *Deladenus bonabensis* n. sp. based on large subunit (LSU) rDNA D2-D3 sequences under GTR + I + G model. Bayesian posterior probability values more than 0.50 are given for appropriate clades. The new sequence is indicated in bold.

Seventy sequences (including the newly generated sequence of the new species and three sequences of classic rhabditids as outgroups) were used in LSU phylogeny (species names and accession numbers are available in the LSU tree). The LSU alignment was composed of 1,023 total characters, of which 796 were variable. Fig. 4 represents the phylogenetic tree inferred using this data set. In this tree, the LSU sequences of *Deladenus* spp. have occupied different placements in the tree, and the the new species has formed a clade with an unidentified sequence (Sphaerularioidea sp., MW577121). The former clade is in sister relation with the LSU sequence of *D. brevis* (MT009494).

## Discussion

During this study, a population of the genus *Deladenus* was recovered from Ghara-Gheshlagh lagoon, Bonab, northwestern Iran. Previously, five species of this genus have been described or reported from Iran (Jahanshahi Afshar et al., 2014; Miraeiz et al., 2017; Esmaeili et al., 2020; Heydari et al., 2020; Jalalinasab et al., 2020). The currently described new species could be conspecific with the previously sequenced species recovered from West Azarbaijan province, of which only three specimens were found (the isolate west.az) and sequenced in our lab (LSU accession numbers MW577121 and MW577120).

Two life cycles including insect parasitic phase and free-living mycetophagous phase are known for *Deladenus* (Siddiqi, 2000). The former species originally added to the genus from Iran were described using their free-living; and in case of *D. gilanica*, two free-living and infective stages. This was the case for the new species, too. A penial tube was observed in males of the new species. This structure is seen in light microphotographs of *Deladenus nitobei* Kanzaki, Tanaka Fitza, Kosaka, Slippers, Kimura, Tsuchiya & Tabata, 2016 as well.

Based on available data, it seems that the genus is not monophylic in both SSU and LSU phylogenies, which is in agreement with previous studies (e.g., Heydari et al., 2020).

## References

[j_jofnem-2023-0002_ref_001] Andrássy I. (1954). Drei neue arten aus der superfamilie Tylenchoidea nematologische notizen3. Annales Biologicae Universitatunz Hungariae.

[j_jofnem-2023-0002_ref_002] Andrássy I. (1957). *Deladenus aridus* n. sp. uad tin Wiedcrfund voa *Deladenus* saccatus Andrássy, 1954. Nemacologischc Notizin 5. Opuscula Zoologica.

[j_jofnem-2023-0002_ref_003] Bajaj H. K. (2015). Further studies on species of *Deladenus* Thorne, 1941 from Haryana, India. Indian Journal of Nematology.

[j_jofnem-2023-0002_ref_004] Chitambar J. J. (1991). On the genus *Deladenus* Thome, 1941 (Nemata: Allantonematidae). Review of the mycetophagous stage. Revue de Nemalogie.

[j_jofnem-2023-0002_ref_005] Cobb N. A. (1922). Two tree-infesting nemas of the genus *Tylenchus*. Anales de Zoología Aplicada.

[j_jofnem-2023-0002_ref_006] De Grisse A. (1969). Redescription ou modifications de quelques technique utilis [a] es dan l’etude des nematodes phytoparasitaires. Mededelingen Rijksfaculteit Landbouwwetenschappen Gent.

[j_jofnem-2023-0002_ref_007] de Man J. G. (1895). Description of three species of Anguillulidae, observed in diseased pseudo-bulbs of tropical orchids. Proceedings and Transactions of the Liverpool Biological Society.

[j_jofnem-2023-0002_ref_008] Dorris M., Viney M. E., Blaxter M. L. (2002). Molecular phylogenetic analysis of the genus *Strongyloides* and related nematodes. International Journal for Parasitology.

[j_jofnem-2023-0002_ref_009] Esmaeili M., Jalalinasab P., Ye W., Heydari R. (2020). Description of *Deladenus gilanica* n. sp. (Hexatylina: Neotylenchidae) isolated from wood of black pine in Northern Iran. Journal of Nematology.

[j_jofnem-2023-0002_ref_010] Fuchs A. G. (1930). Neue an Borken-und Russelkafer gebundene Nematoden, halbparasitische und Wohnungseimieter. Zoologische Jahrbücher, Abteilung für Systematik, Ökologie und Geographie der Tiere.

[j_jofnem-2023-0002_ref_011] Heydari F., Abolafia J., Pedram M. (2020). Description of *Deladenus brevis* n. sp. (Sphaerularioidea: Neotylenchidae) from Iran: A morphological and molecular phylogenetic study. Journal of Nematology.

[j_jofnem-2023-0002_ref_012] Huson D. H., Scornavacca C. (2012). Dendroscope 3: An interactive tool for rooted phylogenetic trees and networks. Systematic Biology.

[j_jofnem-2023-0002_ref_013] Jahanshahi Afshar F., Pourjam E., Kheiri A. (2014). New record of two species belonging to superfamily Sphaerularioidea (Nematoda: Tylenchida) from Iran. Journal of Crop Protection.

[j_jofnem-2023-0002_ref_014] Kanzaki N., Tanaka S. E., Fitza K., Kosaka H., Slippers B., Kimura K., Tsuchiya S., Tabata M. (2016). *Deladenus nitobei* n. sp. (Tylenchomorpha: Allantonematidae) isolated from *Sirex nitobei* (Hymenoptera: Siricidae) from Aomori, Japan, a new member of the *siricidicola* superspecies. Nematology.

[j_jofnem-2023-0002_ref_015] Katoh K., Standley D. M. (2013). MAFFT multiple sequence alignment software version 7: Improvements in performance and usability. Molecular Biology and Evolution.

[j_jofnem-2023-0002_ref_016] Larget B., Simon D. L. (1999). Markov chain Monte Carlo algorithms for the Bayesian analysis of phylogenetic trees. Molecular Biology and Evolution.

[j_jofnem-2023-0002_ref_017] Larkin M. A., Blackshields G., Brown N. P., Chenna R., Mcgettigan P. A., McWilliam H., Valentin F., Wallace I. M., Wilm A., Lopez R., Thompson J. D., Gibson T. J., Higgins D. G. (2007). Clustal W and Clustal X version 2.0. Bioinformatics.

[j_jofnem-2023-0002_ref_018] Miraeiz E., Heydari R., Golhasan B. (2017). A new and a known species of *Deladenus* thorne, 1941 (Nematoda: Neotylenchidae) from Iran, with an updated species checklist of the genus. Acta Zoologica Bulgarica.

[j_jofnem-2023-0002_ref_019] Morris E. E., Stock S. P., Castrillo L. A., Williams D. W., Hajek A. E. (2018). Characterisation of the dimorphic *Deladenus beddingi* n. sp. and its associated woodwasp and fungus. Nematology.

[j_jofnem-2023-0002_ref_020] Mullin P. G., Harris T. S., Powers T. O. (2005). Phylogenetic relationships of Nygolaimina and Dorylaimina (Nematoda: Dorylaimida) inferred from small subunit ribosomal DNA sequences. Nematology.

[j_jofnem-2023-0002_ref_021] Nasira K., Shahina F., Firoza K. (2013). *Deladenus cocophilus* n. sp. (Nematoda: Hexatylina): A mycetophagous and entomoparasitic nematode in infested coconut fruits from Balochistan, Pakistan. Journal of Nematology.

[j_jofnem-2023-0002_ref_022] Nunn G. B. (1992). Nematode molecular evolution: An investigation of evolutionary patterns among nematodes based on DNA sequences.

[j_jofnem-2023-0002_ref_023] Nylander J. (2004). MrModeltest v2. Program distributed by author.

[j_jofnem-2023-0002_ref_024] Poinar G. O. (1986). *Rhabditis myriophila* n. sp. (Rhabditidae: Rhabditida), associated with the millipede, Oxidis gracilis (Polydesmida: Diplopoda). Proceedings of the Helminthological Society of Washington.

[j_jofnem-2023-0002_ref_025] Rambaut A., Drummond A. J. (2009). Tracer version 1.5 (computer program).

[j_jofnem-2023-0002_ref_026] Ronquist F., Huelsenbeck J. P. (2003). MrBayes 3: Bayesian phylogenetic inference under mixed models. Bioinformatics.

[j_jofnem-2023-0002_ref_027] Siddiqi M. R. (2000). Tylenchida: Parasites of plants and insects, 2nd ed.

[j_jofnem-2023-0002_ref_028] Sultavieva G. B. (1983). New species of soil nematodes in Kirgizia. Zoologicheskii Zhurnal.

[j_jofnem-2023-0002_ref_029] Sumenkova N. I. (1975). Nematodes of plants and soil. Neotylenchoidea.

[j_jofnem-2023-0002_ref_030] Tomar V. V., Somvanshi V. S., Bajaj H. K. (2015). Descriptions of *Deladenus albizicus* n. sp. and *D. processus* n. sp. (Nematoda: Hexatylina) from Haryana, India. Journal of Nematology.

[j_jofnem-2023-0002_ref_031] Thorne G. (1925). The genus *Acrobeles* Von Linstow, 1887. Transactions of the American Microscopical Society.

[j_jofnem-2023-0002_ref_032] Thorne G. (1941). Some nematodes of the family Tylenchidae which do not possess a valvular median esophageal bulb. The Great Basin Naturalist.

[j_jofnem-2023-0002_ref_033] von Linstow O. F. B. (1877). Helminthologica. Archiv Für Naturgeschichte.

[j_jofnem-2023-0002_ref_034] Whitehead A. G., Hemming J. R. (1965). A comparison of some quantitative methods of extracting small vermiform nematodes from soil. Annals of Applied Biology.

[j_jofnem-2023-0002_ref_035] Yu Q., Gu J., Ye W., Li R., He J. (2017). *Deladenus posteroporus* n. sp. (Nematoda: Neotylenchidae) isolated from packaging wood from Canada and white pine (Pinus monticola) lumber from the United States and intercepted in Ningbo, China. Journal of Nematology.

[j_jofnem-2023-0002_ref_036] Zell H. (1985). Nematoden eines Buchenwaldboden 4. Die Neotylenchidea (Nematoda, Neotylenchoidea). Carolinea.

